# A multifaceted evaluation of microgliosis and differential cellular dysregulation of mammalian target of rapamycin signaling in neuronopathic Gaucher disease

**DOI:** 10.3389/fnmol.2022.944883

**Published:** 2022-09-20

**Authors:** Zhenting Zhang, Xiaohong Wang, Yi Lin, Dao Pan

**Affiliations:** ^1^Division of Experimental Hematology and Cancer Biology, Cincinnati Children’s Hospital Medical Center, Cincinnati, OH, United States; ^2^Department of Pediatrics, University of Cincinnati School of Medicine, Cincinnati, OH, United States

**Keywords:** Gaucher disease, neurodegenerative disorders, neuroinflammation, mTOR, Lamp1, Tmem119, disease-associated microglia, reactive astrocytes

## Abstract

Neuronopathic Gaucher disease (nGD) is an inherited neurodegenerative disease caused by mutations in *GBA1* gene and is associated with premature death. Neuroinflammation plays a critical role in disease pathogenesis which is characterized by microgliosis, reactive astrocytosis, and neuron loss, although molecular mechanisms leading to neuroinflammation are not well-understood. In this report, we developed a convenient tool to quantify microglia proliferation and activation independently and uncovered abnormal proliferation of microglia (∼2-fold) in an adult genetic nGD model. The nGD-associated pattern of inflammatory mediators pertinent to microglia phenotypes was determined, showing a unique signature favoring pro-inflammatory chemokines and cytokines. Moreover, highly polarized (up or down) dysregulations of mTORC1 signaling with varying lysosome dysfunctions (numbers and volume) were observed among three major cell types of nGD brain. Specifically, hyperactive mTORC1 signaling was detected in all disease-associated microglia (Iba1^high^) with concurrent increase in lysosome function. Conversely, the reduction of neurons presenting high mTORC1 activity was implicated (including Purkinje-like cells) which was accompanied by inconsistent changes of lysosome function in nGD mice. Undetectable levels of mTORC1 activity and low Lamp1 puncta were noticed in astrocytes of both diseased and normal mice, suggesting a minor involvement of mTORC1 pathway and lysosome function in disease-associated astrocytes. These findings highlight the differences and complexity of molecular mechanisms that are involved within various cell types of the brain. The quantifiable parameters established and nGD-associated pattern of neuroinflammatory mediators identified would facilitate the efficacy evaluation on microgliosis and further discovery of novel therapeutic target(s) in treating neuronopathic Gaucher disease.

## Introduction

Gaucher disease, one of the common lysosomal storage disorders (LSDs) with an incidence of 1/800 in the Ashkenazi Jewish population and 1/40,000–1/80,000 in general population, is caused by deleterious mutations in *GBA1* gene encoding acid β-glucosidase (GCase) (Jmoudiak and Futerman, 2005; Grabowski, 2008; Horowitz et al., 2016). Neuronopathic Gaucher disease (nGD) displays neuronal death across the brain and is associated with devastating symptoms in central nerve system (CNS) and premature death. The mutations in *GBA1* gene have been considered as the most common genetic risk factor for Parkinson’s disease (PD) (Riboldi and Di Fonzo, 2019). Neuroinflammation is a hallmark in disease progression of nGD which is characterized by pathological findings such as microgliosis, reactive astrocytosis, and neuron loss (Francelle and Mazzulli, 2022). However, molecular mechanisms leading to neuroinflammation, CNS pathology, and neuron damage and death are not well-understood.

As resident immune cells and the primary mediators of neuroinflammation, microglia play important roles in the pathological outcome of essentially all brain diseases ranging from neurodegenerative disorders, traumatic brain injury, and psychiatric diseases (Wolf et al., 2017). Microglia proliferation in nGD was first implicated in a case report over 60 years ago (Lloyd et al., 1956) and further supported in postmortem samples and animal models by immunohistology analysis or flow cytometry analysis (FACS) in recent years (Kaye et al., 1986; Enquist et al., 2007; Sun et al., 2010; Dasgupta et al., 2015). However, conventional markers for such analyses (e.g., CD68, Mac2, and Iba1) are either short on specificity for microglia or influenced by their activation status that rendering their suitability to assess microglia proliferation independently. Advances in multicolor flow cytometry have made the quantification of microglial subpopulations feasible, yet it is practically challenging in assessing postmortem specimens or samples with small mass. In this study, we first aim to develop a versatile tool by taking advantage of Transmembrane protein 119 (*Tmem119*), a recently identified homeostatic marker specific for resident microglia (Lier et al., 2021), to quantify microglia proliferation and activation independently.

Microglia respond to stressors or CNS insults by polarized activation toward either pro-inflammatory M1 type (neurotoxic) or inflammation-resolving M2 type (neuroprotective) which are associated with unique surface expression patterns and the release of substances, including cytokines, chemokines, and growth factors (Wolf et al., 2017; Kabba et al., 2018; Francelle and Mazzulli, 2022). Recently, multi-dimensional concepts of activation states have been proposed such as disease-associated microglia (Keren-Shaul et al., 2017; Deczkowska et al., 2018) which present partially overlapping sets of intracellular and cell surface markers, and secretory products (Wolf et al., 2017; Dubbelaar et al., 2018; Cho et al., 2019; Prinz et al., 2019). Other than heterogeneous microglial populations, reactive astrocytes (neuroprotective or neurotoxic) also contribute to expression and response of different inflammatory mediators. Thus, each neurological LSD has its own disease-associated pattern of neuroinflammation, being chronic or acute, due to different accumulation of storage material (Vitner et al., 2016). The second objective of the study was to determine nGD-associated pattern/signature of inflammatory mediators pertinent to microglia phenotypes to shred some lights on CNS disease pathogenesis in neuronopathic Gaucher disease.

Dysfunctions of lysosomes in glia and neurons play important roles in neurological LSDs pathogenesis, in part, via their close relationship with the mammalian target of rapamycin complex 1 (mTORC1) signaling pathway, a master regulator of cell growth and metabolism (Walkley, 2021). mTORC1 activity is tightly controlled by its recruitment to the lysosomal surface for intimate proximity to the activating GTPase and sensing nutrients (Liu and Sabatini, 2020). On the contrary, hyperactivity of mTORC1 signaling is found to reduce lysosome biosynthesis. Previous studies revealed hypoactive (downregulated) mTORC1 in the head of *Gba1*-deficient *Drosophila* models and peripheral blood mononuclear cells (PBMC) from GD patients (Kinghorn et al., 2016; Ivanova et al., 2019). Conversely, hyperactive (upregulated) mTORC1 signaling has been reported either in neuronal cells derived from induced pluripotent stem cells (iPSC) of nGD patients with reduced lysosome function (Brown et al., 2019), or in an immortalized neuronal cell line derived from a *Gba* knockout (*Gba^–/–^*) mouse model with upregulated lysosome function (Peng et al., 2021). Thus, we investigated mTORC1 signaling and lysosome function in brain tissues by Western blot analysis or RT-qPCR and evaluated *in situ* the correlation of mTORC1 activity and lysosome puncta in microglia and astrocytes, resting or activated, as well as neurons by immunofluorescent (IF) analysis in 4L;C* mice, a genetic model representing type 2/3 nGD (Sun et al., 2010).

In this study, we develop a convenient and efficient tool to quantify microglia proliferation and activation independently and uncover a ∼2-fold increase in microglia of nGD mice. In addition, we investigate nGD-associated pattern of inflammatory mediators and evaluate *in situ* the correlation of mTORC1 activity and lysosome function in microglia, astrocytes and neurons. Collectively, our findings suggest that pro-inflammatory mediators dominate nGD-associated neuroinflammation, with chemokine Cxcl10 escalating the highest. Robust upregulation of both mTORC1 signaling and lysosome puncta is found in nearly all activated microglia, but hypoactive mTORC1 detected in neurons (especially Purkinje neurons) is apparently not associated with consistent alteration of lysosome function. mTORC1 signaling may play a minor role in disease-associated reactive astrocytosis. More detailed molecular and neuropathological studies focused on specific inflammatory pathways and mTORC1 signaling pathways should provide insight into the role of inflammation and mTORC1/lysosome dysfunctions in the progression of nGD.

## Materials and methods

### Animals

C57BL/6J strain of 4L;C* mice were used which carry mutated GCase V394L (4L in short) and deficient saposin C. They were generated by cross-breeding of 4L homozygote into the saposin C-null (C^–/–^) mouse. The 4L;norm (homozygote for 4L allele, WT, or heterozygote for saposin C allele) littermates have no apparent peripheral and CNS abnormalities and are used as normal controls in experiments. All animal procedures were performed in a pathogen-free facility at the Cincinnati Children’s Research Foundation (CCRF) in the vivarium fully accredited by Association for the Assessment and Accreditation of Laboratory Animal Care and approved by Institutional Animal Care and Use Committee at CCRF.

## Results

### Abnormal proliferation and activation of microglia found in 4L;C* brain can be evaluated quantitatively and independently

**FIGURE 1:**
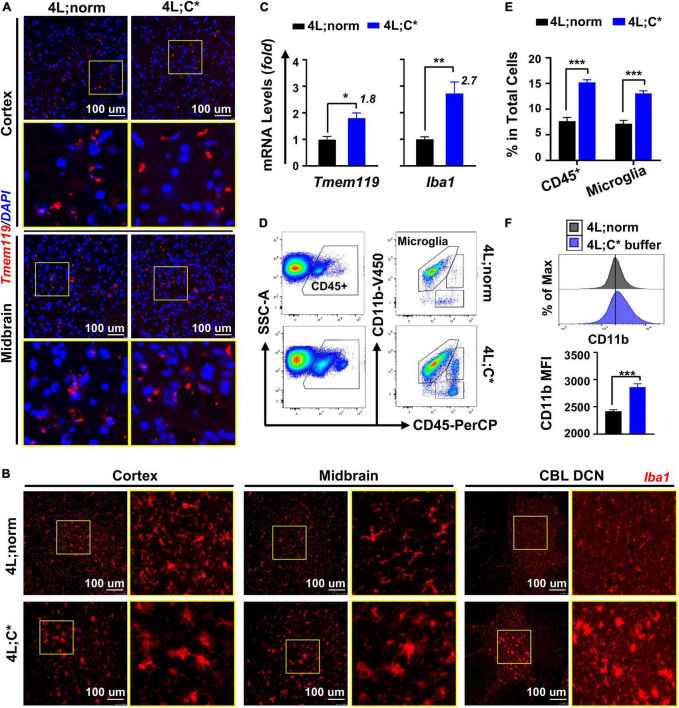
Microglia showed abnormal elevation in both the numbers and activation status in 4L;C* brain. **(A,B)** Immunofluorescent analysis for microglial cells with anti-Tmem119 (red in **A**) or anti-Iba1 (red in **B**) and DAPI for nucleus (blue). **(C)** Relative mRNA expression of *Tmem119* and *Iba1* by RT-qPCR analysis using midbrain samples. *N* = 4 for each group. **(D)** Representative dot plots of FACS analysis with staining for microglia (CD11b^+^CD45^intermediate^) using cells isolated from brain hemispheres. **(E)** Quantitative analysis of CD45^+^ cells and microglia in total mononuclear cells obtained from half brain. **(F)** Mean florescent intensity (MFI) for CD11b expression in microglial cells. Representative histogram plots are shown (top) with quantitative analysis in each group (bottom). *N* = 12–18 for each group in **(E,F)**. Data are presented (in **C,E,F**) as mean ± SEM. *p ≤ 0.05, **p ≤ 0.01, and ***p ≤ 0.001 by Student’s *t*-test.

Tmem119 has recently been identified as a homeostatic marker specific for resident microglia (Lier et al., 2021). However, it is not clear whether Tmem119 expression would vary in activated or quiescent microglia of the nGD model. Immunofluorescence analysis with anti-Tmem119 antibody showed comparable Tmem119 distribution with similar signal intensities in the brain of 4L;C* as those of control mice (Figure 1A). Conversely, IF staining for the expression of Iba1, a well-known microglia/macrophage-specific calcium-binding protein, showed more intensified signals in the diseased brain, which identified either activated microglia or brain macrophages recruited from peripheral blood with significantly enlarged cell body and less ramified morphology (Figure 1B). The observations demonstrated the unaffected expression of Tmem119 in microglia of nGD mice, regardless of resting or activated states. The data support the suitability of Tmem119 as a microglial marker that is directly correlated to the numbers of microglial cells (separately from activation status) in nGD.

We then assessed the degrees of abnormal growth in microglia by RT-qPCR analysis. The quantification of *Tmem119* mRNA expression showed 1.8-fold increase in the brain of 4L;C* mice as compared to 4L;norm controls, demonstrating aberrant microglial proliferation in nGD mice (Figure 1C). The expression levels of *Iba1* exhibited a higher degree (2.7-fold) of elevation than those of *Tmem119* in 4L;C*, supporting the notion that alteration of Iba1 expression is associated with the increase in both the numbers and the activation status of microglial cells as observed in IF analysis (Figure 1B). Moreover, the differential increase between *Tmem119* and *Iba1* may serve as a measurable parameter specifically for microglial activation.

To validate the relevance of *Tmem119*-targeted assessment and confirm the degrees of microglia proliferation and activation in nGD, we analyzed brain mononuclear cells isolated from hemispheres of well-perfused 4L;C* mice and healthy littermates (4L;norm) by flow cytometry. The success of perfusion to eliminate blood contamination was confirmed in each animal by the loss of color in the liver and blood vessels that flank the midline of the rib cage. A two-step gating strategy was utilized by immunofluorescent staining for CD45 (a pan-hematopoietic marker) and CD11b (also known as integrin alpha M, subunit of Mac-1) (Figure 1D). Differential CD45 expression separated resident microglia (CD45^intermediate^) from migrated blood cells (CD45^high^) and non-hematopoietic cells, and CD11b further refined microglial cells (CD11b^+^CD45^intermediate^). The percentages of total microglial cells in 4L;C* were significantly increased by 1.8-fold as compared to 4L;norm controls (Figure 1E). The mean fluorescence intensity (MFI) of CD11b in total microglial population was significantly elevated in 4L;C* as compared to 4L;norm, confirming its suitability to assess activation levels of microglia in nGD (Figure 1F). Importantly, the data confirmed the 1.8-fold increase in microglia proliferation as determined by RT-qPCR in 4L;C* mice (Figure 1C). Taken together, the results document not only that the expression of *Tmem119* was not responsive to pathological microglial activation in nGD mice, but also mRNA expression of *Tmem119* by RT-qPCR is validated as a convenient and effective marker/tool to assess microglial proliferation quantitatively and independently.

### Disease-associated neuroinflammatory pattern pertinent to microglia presents a unique signature favoring pro-inflammatory mediators

**FIGURE 2:**
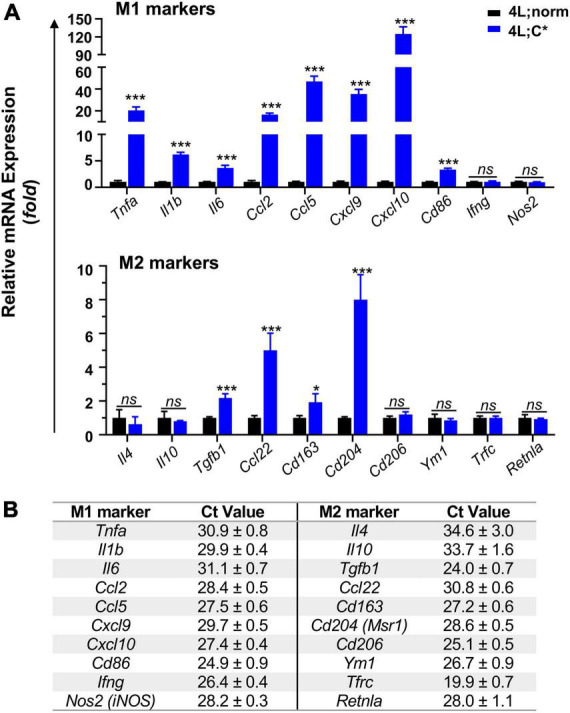
Relative mRNA expression and abundance of selected M1/M2 makers in the brain of mice harvested at ∼55 days of age. Middle brain part (cerebrum without cortex and brain stem) was used for mRNA isolation and RT-qPCR analysis using 5 ng mRNA. **(A)** Expression levels of main M1 and M2 markers are shown with *Tbp* used as internal reference gene. Data are presented as mean ± SEM, *n* = 4. *p ≤ 0.05; ***p ≤ 0.001; ns, non-significant by Student’s *t*-test analysis. **(B)** Baseline Ct values of M1/M2 markers in 4L;norm control samples, showing relative mRNA abundance (with *Tbp* as 22.2 ± 0.3). Data are presented as mean ± SD.

To determine nGD-associated transcriptional patterns correlated with disease-associated microglia in 4L;C*, we investigated mRNA expression of markers for M1 and M2 microglia which were often found aberrant in neurodegenerative diseases (Tang and Le, 2016; Guo et al., 2022). For pro-inflammatory M1 markers, we observed significant increase for most candidates in 4L;C* mice as compared to healthy 4L;norm controls (Figure 2A). Cytokines *Tnfa* exhibited the highest elevation (20-fold) and were followed by *Il1b* (6-fold) and *Il6* (4-fold). Among the chemokines, *Cxcl10* showed the most dramatic change with 125-fold increase. *Ccl2*, *Ccl5*, and *Cxcl9* were also significantly elevated by 17-, 47-, and 35-fold, respectively. In addition, antigen-presenting cell receptor *Cd86*, which was most abundant among all M1 markers tested (Figure 2B) and a cell surface protein identifying M1 microglia (Mills Ko et al., 2014), was also increased by 3-fold. Interestingly, no significant alteration was observed for *Nos2* and *Ifng* which triggers type II interferon (IFN)-mediated signaling. The data demonstrated that most of the M1 makers tested showed either robust (> 20-fold in 5/10 candidates) or moderate increase (3–6 fold in 3/10 candidates) in 4L;C* compared to normal littermates.

Most anti-inflammatory M2 markers tested showed either no or moderate (≤8-fold) disease-associated changes, with 2-fold elevation for neuroprotective cytokine *Tgfb1* and 5-fold for chemokine *Ccl22* (Figure 2). Scavenger receptors *Cd163* and *Cd204* were also increased by 2-fold and 8-fold, respectively. However, *Cd206* and *Ym1*, transmembrane proteins for M2 microglia identification, showed similar expression levels in 4L;C* and healthy controls. No significant variations were also observed for all other M2 markers, including cytokines *Il4* and *Il10* as well as *Tfrc* and *Retnla*. These results showed only a portion of M2 markers (4/10) were significantly elevated (by only 2–8 fold). The data presented a unique nGD-associated pattern favoring pro-inflammatory mediators with *Cxcl10* elevating the most in an adult genetic model of neuronopathic Gaucher disease.

### Hyperactivity of mammalian target of rapamycin complex 1 signaling is detected in disease-associated microglia and brain tissues with regional variations, while high lysosomal Lamp1 is also found in many activated microglia of neuronopathic Gaucher disease

**FIGURE 3:**
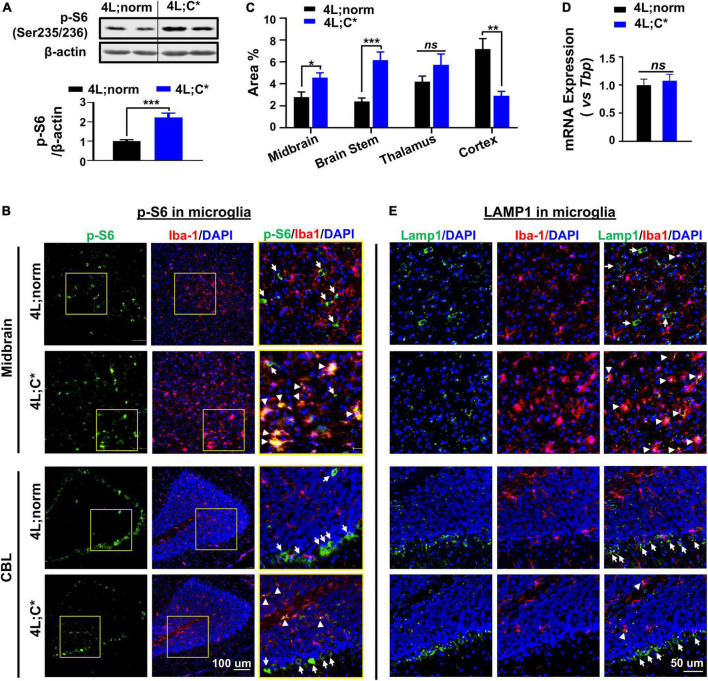
Diverse expression of p-S6 and Lamp1 in microglia and brain tissues. **(A)** Protein levels of p-S6 (direct downstream target of mTORC1) were abnormally elevated in 4L;C* brain. Representative immunoblots of brain stem tissues for p-S6 (Ser235/236) are shown (top panel) with semi-quantitation of signal intensity by Image J (bottom panel). Data are presented as mean ± SEM, *n* = 6–9 per group. ***p ≤ 0.001 by Student’s *t*-test. **(B)** Representative views of p-S6 signals in midbrain and CBL regions. Areas highlighted in yellow squares were enlarged and shown in right panel. Strong p-S6 signals are indicated for co-localization with (white triangles) or without (white arrows) Iba1 signals. **(C)** Semi-quantitative analysis of overall p-S6 signals in different cerebral regions of 4L;C* and controls. Data are presented as mean ± SEM, with 4–7 images analyzed for each region of each animal with two mice per group. *p ≤ 0.05, **p ≤ 0.01, and ***p ≤ 0.001, ns, non-significant by Student’s *t*-test. NIS-Elements AR software was used for image analysis. **(D)** Relative mRNA expression of *Lamp1* in brain. Data are presented as mean ± SEM, *n* = 4. ns, non-significant by Student’s *t*-test. **(E)** Representative views of Lamp1 signals. Strong Lamp1 signals are indicated for co-localization with (white triangles) or without (white arrows) Iba1 signals.

Inconsistent observations have been reported on dysregulation of mTORC1 in nGD studies depending on the models and cell lines evaluated (Dasgupta et al., 2015; Kinghorn et al., 2016; Brown et al., 2019; Ivanova et al., 2019; Peng et al., 2021; Srikanth et al., 2021). The potential *in vivo* alteration of mTORC1 activity was determined by Western blot analysis in brain tissues from genetic nGD model and healthy littermates (Figure 3A). Phosphorylated ribosomal protein S6 (Ser235/236) (p-S6) is a direct target and common marker for mTORC1 activity (Marosvári et al., 2018). The p-S6 protein levels were significantly increased (2.2-fold) in the brain stem of 4L;C* mice, suggesting hyperactivity (upregulation) of mTORC1 pathway.

To pinpoint dysfunctions of mTORC1 signaling in different types of brain cells and different brain regions, we evaluated p-S6 expression in microglial cells by IF staining with microglia marker Iba1. Widespread p-S6 signals were observed across the cerebrum and cerebellum (CBL) of 4L;norm and 4L;C* mice, including midbrain, brain stem, thalamus, cortex, and deep cerebellar nuclei regions (Figure 3B and Supplementary Figures 1A,B). Semi-quantitative analysis of immunofluorescent p-S6 signals revealed an overall increase in p-S6 levels in the midbrain (1.7-fold), brain stem (2.6-fold), and thalamus (1.4-fold) regions of 4L;C* as compared to 4L;norm controls. However, in the cortex region, the diseased mice exhibited significantly less p-S6 signals (∼40% of normal levels) than those of 4L;norm controls (Figure 3C). These observations indicate high regional variations on overall p-S6 expression. Further evaluation of p-S6 signals in Iba1^+^ microglia demonstrated that, in 4L;norm controls, almost none of p-S6 signals were correlated with Iba1^+^ microglia, suggesting generally low mTORC1 activity in microglial cells. Conversely, in the diseased 4L;C* mice, highly intensified p-S6 signals were detected in nearly all activated microglia (with enlarged cell body) as evidenced by high co-localization of p-S6 with Iba1 signals in all brain regions assessed (Figure 3B and Supplementary Figures 1A,B). Similarly in cerebellum region, strong but non-microglia associated p-S6 signals (particularly high in Purkinje-like cells) were observed in 4L;norm, while p-S6 signals were highly co-localized with microglia and fewer Purkinje-like cells in 4L;C* mice (Figure 3B). Taken together, these observations demonstrated that the p-S6 signals in microglia, which were barely detectable under normal physiological condition, were significantly upregulated in almost all activated microglia of 4L;C* mice. The data suggest that mTORC1 pathway is upregulated in nGD-associated activated microglia, with varied overall p-S6 signals among different brain regions.

Activation of mTORC1 is dependent upon its recruitment to the lysosomal surface (the site of activation) (Liu and Sabatini, 2020). We then evaluated possible alteration of lysosome-associated protein 1 (Lamp1) for potential dysregulations of lysosomes. The expression levels of *Lamp1* mRNA were similar in the brain of 4L;C* as those of healthy littermates, suggesting no overall changes in concurrent Lamp1 generation (Figure 3D). Interestingly, the LC3B-II protein levels were abnormally elevated in the diseased brain as compared to normal controls, indicating aberrant lysosomal-autophagy metabolism (Supplementary Figure 2). Further IF analysis showed comparable overall levels of Lamp1 puncta, but with highly distinct distribution patterns among different cells, across cerebrum and cerebellum of 4L;C* as compared to 4L;norm controls (Figure 3E and Supplementary Figure 1A). Lamp1 puncta are attributable to both the numbers and volume of lysosomes. In healthy littermates, Lamp1 puncta were mostly associated with non-microglial (Iba1^–^) cells and were almost undetectable in Iba1^+^ microglia except for few activated microglial cells across the brain. However, in 4L;C* mice, strong and condensed Lamp1 signals were observed specifically in activated microglia which were identified by intense Iba1 signals. Interestingly, the Purkinje-like cell layers in CBL of 4L;C mice showed similar levels of Lamp1 signals as those of 4L;norm mice (Figure 3E). Of note, a few cells were found to express strong Iba1 but no Lamp1 in both normal and diseased brains, implying either migrated brain macrophages or alternative microglia state regardless of physiological or disease condition. These results suggested that most nGD-associated, activated microglial cells exhibited upregulation of lysosomal function, even though hyperactive mTORC1 signaling detected in these cells (Figure 3B) is commonly correlated to repression of lysosome biosynthesis.

### Downregulation of mammalian target of rapamycin complex 1 activity is observed in neurons of neuronopathic Gaucher disease mice, which has no consistent association with Lamp1 signals

**FIGURE 4:**
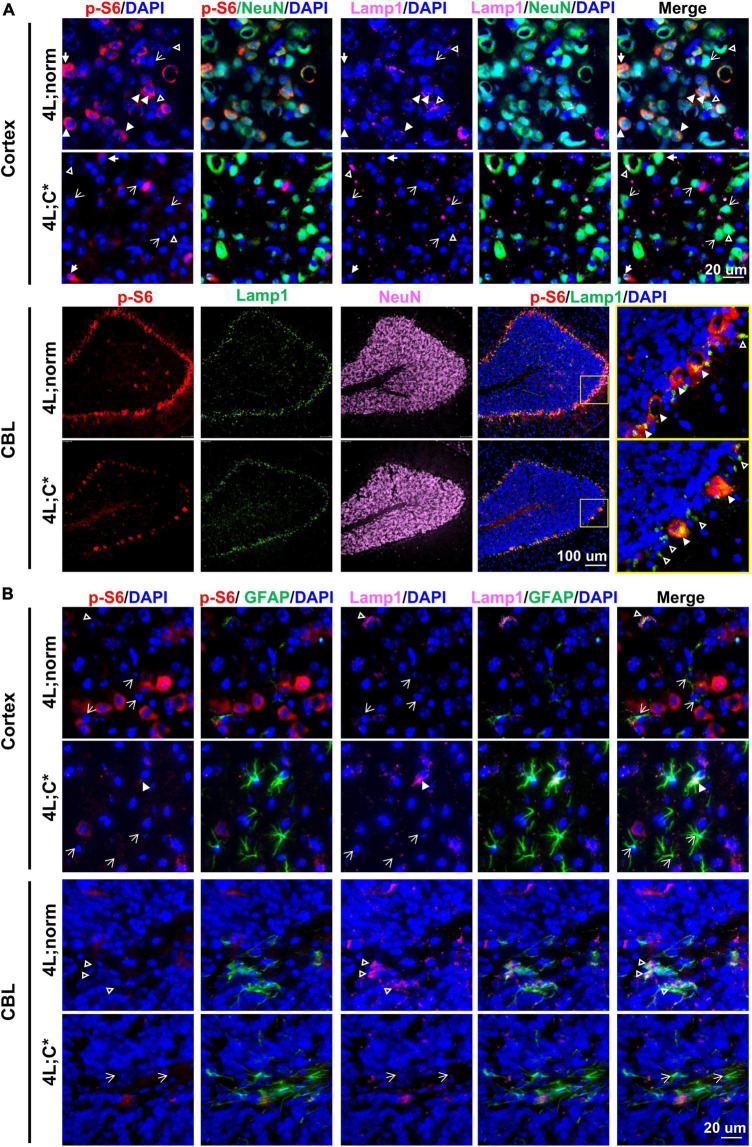
Expression of p-S6 and Lamp1 in neurons and astrocytes. Representative views of p-S6 and Lamp1 signals in neurons **(A)** or astrocytes **(B)** of cortex and CBL regions. In **(A)**, neurons (or astrocytes in **B**) with detectable Lamp1 are indicated for co-localization with (white triangles) or without (open triangles) strong p-S6 signals; neurons (or astrocytes in **B**) with undetectable Lamp1 are indicated either with (solid arrows) or without (open arrows) p-S6 expression.

Potential dysfunction of mTOR activity and its association with Lamp1 expression in neurons were examined by four-color IF analysis with anti-NeuN antibody in cortex, midbrain, and thalamus regions and cerebellum. In healthy controls, the expression of p-S6 in NeuN^+^ neurons varied dramatically with some exhibiting intense signals and some showing low or barely detectable expression (Figure 4A and Supplementary Figure 3). However, 4L;C* mice presented much less p-S6 signals in NeuN^+^ neurons (that is, much fewer p-S6^high^NeuN^+^ neurons across the cerebrum), implying that mTORC1 activity was significantly reduced in some neurons which may be associated with disease pathology. Moreover, the lobe-shape, Purkinje-like cell layer (NeuN-negative) in cerebellum of 4L;C* mice showed significantly less p-S6 signals when compared with those in 4L;norm controls (Figure 4A). Purkinje neurons play pivotal roles in coordination, controlling and learning of movements. Thus, the marked downregulation of mTOR activity in Purkinje cells may likely contribute to motor function deficits in nGD patients.

For Lamp1 expression in neurons, no significant difference was observed between 4L;C* and 4L;norm mice (Figure 4A and Supplementary Figure 3). Interestingly, there seemed to have no significant correlation of Lamp1 signals with p-S6 signals in NeuN^+^ neurons of either nGD or normal controls across the cerebrum. Such apparent disengagement was also consented by the observation in cerebellum where Lamp1 signals in Purkinje-like cell layer were comparable in both 4L;C* and controls in spite of much less p-S6 signals detected in diseased mice (Figure 4A). These results suggested that neuronal lysosome dysfunction (the number and volume) as defined by Lamp1 puncta would likely involve other mechanisms, in addition to mTOR-dependent lysosome biosynthesis.

### Neglectable levels of mammalian target of rapamycin complex 1 activity is observed in astrocytes regardless of activation status or disease condition

We evaluated mTORC1 activity and its association with lysosome compartment in astrocytes by four-color IF analysis with anti-GFAP antibody across the brain. Significantly more intense GFAP signals were found across different regions of cerebrum and the granule cell layers of cerebellum in 4L;C* than those in 4L;norm controls (Figure 4B and Supplementary Figure 4). Apparently, no p-S6 signals were detected in most GFAP^+^ astrocytes, although strong Lamp1 signals were detected sporadically and co-localized with GFAP signals across cerebrum and white matter of cerebellum in both diseased and normal mice. The data suggested that mTORC1 signaling may play a minor role in disease-associated reactive astrocytosis.

### Discussion

Microgliosis has been reported in postmortem nGD patients (Wong et al., 2004; Burrow et al., 2015) and nGD mouse models (Enquist et al., 2007; Farfel-Becker et al., 2011) based on signal intensity and frequency by immunostaining analyses for Iba1, Mac2, and CD68. Both abnormal proliferation and activation in microglia are indicated, each may be correlated with different molecular mechanisms and cellular processes in disease progression. For example, significant increase in CD68^+^ cells was detected in chemical-induced nGD model (Marshall et al., 2016) or 4L;C* mice (Sun et al., 2010) with early onset of CNS manifestations. However, comparable levels of CD68^+^ expression were observed in a chronic nGD model and normal controls (Dai et al., 2016). In addition, these markers are associated with either activated microglia or migrated brain macrophages (both are elevated in nGD), thus are not decisive to assess potential variations in total numbers of microglial cells (proliferation). Tmem119 has been reported to be IHC-positive in microglia but not in peripheral immune cells (Bennett et al., 2016) and was used to distinguish resident microglia from migrated peripheral cells in Alzheimer’s disease (Satoh et al., 2016) and glioma (Haage et al., 2019). Importantly, consistent levels of Tmem119 were observed regardless of pathological status in both cases. In neurodegenerative nGD brain, the intensities of Tmem119 were similar as those in normal controls; whereas much stronger signals of Iba1 were detected in activated microglia with morphological changes in 4L;C* brain than those in healthy brain. Thus, we confirmed Tmem119 as a ubiquitously expressed microglia marker that is not associated with pathological microgliosis in nGD.

Moreover, we attested quantitatively that microglial proliferation increased by 1.8-fold in 4L;C* mice as compared to age-matched normal controls using RT-qPCR analysis for mRNA expression of *Tmem*119. Such degrees of increase were confirmed by multicolor FACS analysis for CD45^intermed^CD11b^+^ cells in brain mononuclear cells isolated from half of the brain. The levels of microglial activation in nGD mice were measured by quantifiable parameters, including the differential changes of mRNA expression between activation-associated *Iba*1 (2.7-fold) and activation-unrelated *Tmem*119 (1.8-fold) and MFI of CD11b expression in microglial population by FACS assay. The data not only demonstrate unambiguously the doubling of resident microglial cells in nGD mice, but also provide a convenient and efficient tool to assess the proliferation and activation of microglial cells quantitatively and independently.

Under pathological conditions, microglia are activated, migrate to the injury site, engulf debris, sense pathology, and secrete pro-inflammatory and/or anti-inflammatory factors to either exacerbate or offset disease progression (Ho, 2019). Microglia from each disease-associated state express a unique transcriptional signature (Keren-Shaul et al., 2017; Krasemann et al., 2017). Inflammation in peripheral organs of GD, thought to be triggered by substrate accumulation in macrophages, has been documented with characteristic elevation of cytokines including IFNγ, TNF-α, and IL-1β and overactivation of the immune system (Pandey and Grabowski, 2013; Pandey et al., 2017). In adult 4L;C brain, we observed no changes in the expression of IFNγ which is key component in type II IFN response. Moreover, significant enrichment of genes involved in type I IFN response was reported in pre-symptomatic nGD mice (*Gba*^flox/flox^; nestin-Cre model) (nGD^nestin^) (Vitner et al., 2016); however, genetic blockage of IFN response introduced no survival benefits in chemical-induced nGD mice (Vardi et al., 2020). Thus, both IFN pathways may play secondary or insignificant role in nGD neuropathogenesis.

The beneficial or detrimental effects of microglial cells to brain health might be dependent on several factors, including the nature and magnitude of the initial insults and the levels and time of immune mediator release. Thus, the patterns of neuroinflammation on characteristic inflammatory mediators/factors are disease-associated and may vary during different developmental stages and/or disease progression. It is noticeable that the upregulation magnitudes and the orders of main pro-inflammatory cytokines found in 4L;C* brain, that is, *Tnfa* > *Il1b* > *Il6* (20-, 6-, and 4-fold), were distinct from those reported in another genetic nGD^nestin^ mice, that is, *Il6* > *Tnfa* > *Il1b* (22-, 11-, and 7.5-fold) (Vitner et al., 2016). Elevated expression and secretion of TNF-α from activated microglia are believed to facilitate conversion of astrocytes into cytotoxic subtype and play a direct role in PD and AD (Liddelow et al., 2017), whereas exogenous IL1β has been reported to stimulate proliferation and reactivity of microglia (Todd et al., 2019).

Among chemokines, relatively milder elevation was found in 4L;C* model (with 47-fold for *Ccl5* and 17-fold for *Ccl2*) as compared to those in nGD^nestin^ mice (with 240-fold for *Ccl5* and 129 for *Ccl2*) (Vitner et al., 2016). Interestingly, *Cxcl10* (125-fold) was the most upregulated inflammatory mediator (among comparable set of genes tested) in 4L;C* mice, with *Ccl5* (240-fold) as the most elevated mediator in nGD^nestin^ mice and *Ccl2* (161-fold) in the chemically induced nGD. Several reasons may have contributed to such significantly diverged patterns. First, unlike 4L;C* mice with global GCase deficiency in all cells, microglial cells in nGD^nestin^ (Enquist et al., 2007) expressed normal *Gba1* and their activation is induced only by extrinsic stimuli from other brain cells. Molecular pattern in chemically induced nGD model would be complicated by chemical-associated off-target effects, change of brain penetration with age, and simplified/altered pathways at cellular levels (such as protein misfolding or intro-cellular trafficking) as compared to genetic nGD models. Second, different brain developmental stages (adolescent vs. adult) may influence neuropathogenic molecular signatures due to altering functions (on demands) of microglia during developing or maintenance stages. Finally, varying specimen regions (cerebral hemisphere vs. midbrain) may play a role due to differences in regional distribution of various cell types and cell-specific pathogenic processes. It should be recognized that disease-associated molecular patterns are model-sensitive and context-specific. Further comprehensive studies would be needed to delineate the roles of different pathways in the neuroinflammation.

The reports on how mTORC1 signaling pathway is dysregulated and involved in CNS pathogenesis vary considerably among different LSDs and often diverge in opposite ways in different organs, cell types, or experimental conditions. Hypoactive mTORC1 signaling was observed in the head of *Gba1*-deficient *Drosophila* models and peripheral blood mononuclear cells from GD patients (Kinghorn et al., 2016; Ivanova et al., 2019). This is consistent with downregulation of mTORC1 activity reported in the brain of Niemann-Pick type C, another neurodegenerative disease (Zervas et al., 2001). However, we reported an overall elevation of mTORC1 signaling (2.2-fold) in 4L;C* brain by Western blot analysis for p-S6 protein. This observation is in agreement with previous finding, showing increased mRNA expression in several genes on mTOR signaling pathway in the brain of 4L;C* mice (Dasgupta et al., 2015).

Divergent and cell type-specific dysregulation of mTOR signaling was documented in this study, with hyperactive mTORC1 signaling found in abnormally activated microglia (Iba1^high^) but hypoactivity in neurons (or reduction of p-S6^high^ neurons) of nGD mice. Generally, low mTORC1 activity found in all microglia of healthy littermates with undetectable p-S6 signals was markedly upregulated in all activated microglial cells (Iba1^high^) 4L;C*, indicating hyperactive mTORC1 signaling in disease-associated microglia. It has been reported that damage-associated molecular pattern (like ATP) activated mTORC1 activity and increased synthesis of pro-inflammatory cytokines in microglia (Hu et al., 2020). Further inhibition of mTORC1 hyperactivity by rapamycin significantly reduced the abnormally elevated production of pro-inflammatory cytokines (such as TNF-α), suggesting that mTORC1 signaling plays an important role in regulating cytokine expression in microglia (Hu et al., 2020). On the contrary, exposing microglial cells with a pro-inflammatory cytokines mixture (including TNF-α and Il-1β) could trigger a full activation of microglia and hyperactive mTORC1 (Dello Russo et al., 2009). With continuous stress from intracellular and intercellular stimuli within glia and neurons of diseased brain, persistent elevation of pro-inflammatory mediators may further activate microglia and upregulate mTORC1 activity to exacerbate neuroinflammatory conditions, leading to neuron loss and CNS manifestations in patients and mouse models with nGD.

Importantly, reduction of neurons with high mTORC1 activity was shown across the brain of 4L;C* mice as demonstrated by the loss of p-S6 signals in many neurons, including Purkinje cells in the cerebellum. This observation is not in support of mTORC1 hyperactivity found in neurons derived from iPSC of nGD patients or neuronal cell line derived from *Gba*^null^ mice (Brown et al., 2019; Peng et al., 2021; Srikanth et al., 2021). Such deviating observations in neuronal mTORC1 dysfunction may be attributable to the sensitive nature of mTORC1 pathway to metabolic cues under brain microenvironment or culture conditions. It was noticeable in normal controls that neurons with high mTORC1 activity were observed either well-mixed with neighboring low-activity neurons or concentrated in certain regions (such as Purkinje cells). The hypoactive mTORC1 pathway detected in nGD neurons may display a disease-associated status, such as apoptosis or necroptosis, within individual neuron. Alternatively, it may represent an unbalanced or irreversible shift from “hyperactive” neurons converging into “hypoactive” neurons. Of note the 4L;C* mice were all active and in fair health at the time of experiment (6 days prior of average lifespan), although full spectrum of CNS functional deficits was detected at this age (data not shown). A detailed *in vivo* evaluation of mTORC1-hypoactive neurons under healthy or nGD condition may provide some insights on molecular and cellular mechanisms involved in CNS disease pathogenesis.

Lysosome dysfunctions determined by Lamp1 puncta, a readout affected by both the size/volume and the numbers of lysosomes, represent an overall outcome from several cellular processes. Generally, active mTORC1 signaling negatively controls (attenuate) lysosomal biogenesis by inhibition of transcriptional factor TFEB (Laplante and Sabatini, 2012). This is consistent with the report that hyperactive mTORC1 was correlated with significant reduction in TFEB-mediated lysosome biogenesis in iPSC-derived neuronal cells from nGD patients (Brown et al., 2019). In this study, however, we found increased lysosomal Lamp1 puncta in most of abnormally activated microglial cells which also presented hyperactive mTORC1 signaling. Moreover, the apparent disassociation of Lamp1 signals with mTORC1 activity in neurons, such as that, no significant changes in Lamp1 puncta were observed in Purkinje-like cell layers which were accompanied with substantial loss of mTORC1^high^ cells in nGD mice, also supports this notion. In addition to mTORC1-dependent lysosomal biosynthesis, other factors may also contribute (offset) to lysosome dysfunctions (presented by Lamp1 puncta) in different types of brain cells of nGD, including abnormal storage material in lysosomes, dysfunctions in autophagosome/lysosome/endosome networks, and potential disturbance in the recycling of lysosomes from autolysosomes. The association of hyperactive mTORC1 with upregulated lysosomal Lamp1 puncta in activated microglia we observed suggested that dysfunctions in mTORC1-independent cellular mechanisms may play major roles in nGD-associated microglia and need to be further investigated.

Inhibition of mTORC1 by rapamycin (or its derivatives) has been shown to provide neuroprotective benefits in animal models of neurodegenerative diseases, such as multiple sclerosis, Alzheimer’s disease, and Parkinson’s disease (Bove et al., 2011; Lin et al., 2017; Chen et al., 2019; Li et al., 2020). However, rapamycin was reported to decrease the survival of GD iPSC-derived neuronal cells which were associated with hyperactive mTORC1 signaling *in vitro* (Awad et al., 2015). Interestingly, in GCase-deficient *Drosophila* model which presented hypoactive mTORC1 in the head, treatment of rapamycin resulted in significant improvement of lifespan and movement phenotypes (Kinghorn et al., 2016). Similar to the fly model, we found hypoactive mTORC1 in neurons of diseased mice, although upregulated mTORC1 signals were observed in brain tissues which were likely derived from hyperactivity in activated microglia. Thus, it would be important to explore potential CNS benefits of pharmaceutical mTORC1 inhibitors for the treatment of nGD.

## Conclusion

The profiling study focusing on neuroinflammatory mediators related to neurotoxic and neuroprotective microglia has described a unique disease-associated pattern in an adult genetic model of neuronopathic GD. The pathological microgliosis in 4L;C* mice was accompanied with ∼2-fold microglia proliferation using a newly developed tool for Tmem119 expression. Polarized (up or down) dysfunctions of mTORC1 activity and their association with lysosome puncta were documented in the three major cell types of the brain, including microglia showing hyperactive mTORC1 signaling coinciding with increased lysosome function, neurons with increasing numbers of mTORC1-hypoactive neurons without consistent alteration in lysosome function, and reactive astrocytes with neglectable background signals. These findings highlight the differences and complexity of molecular mechanisms that are involved within various types of brain cells. The quantifiable parameters established and nGD-associated pattern of neuroinflammatory mediators identified would facilitate the efficacy evaluation on microgliosis and further discovery of novel therapeutic target(s) for the treatment of neuronopathic Gaucher disease.

## Data availability statement

The raw data supporting the conclusions of this article will be made available by the authors, without undue reservation.

## Ethics statement

This animal study was reviewed and approved by the Institutional Animal Care and Use Committee at Cincinnati Children’s Research Foundation.

## Author contributions

DP conceived the study, obtained financial support, and was responsible for project management. ZZ, XW, and YL conducted the experiments. ZZ, XW, YL, and DP analyzed the data. ZZ and DP generated the figures and wrote the manuscript. All authors contributed to the article and approved the submitted version.
