# Supplementary material for: A multifaceted evaluation of microgliosis and differential cellular dysregulation of mammalian target of rapamycin signaling in neuronopathic Gaucher disease
Source: Front Mol Neurosci. 2022 Sep 20;15:944883. doi: 10.3389/fnmol.2022.944883 (PMC9530712; doi:10.3389/fnmol.2022.944883)
Supplement: Supplementary file 1 [file Data_Sheet_1.pdf]

Supplementary Method and Material

Animal Perfusion

4L;C* and 4L;norm control were harvested at ~55 days of age. Under deep anesthesia with sodium pentobarbital (40 mg/kg) (Abbott Laboratories, Chicago, IL, USA), each mouse underwent trans-cardiac perfusion with sterile, ice-cold phosphate-buffered saline (PBS) for 5 min. The success of this procedure was confirmed by a loss of color in the liver and the blood vessels that flank the midline of the rib cage. The brain was then quickly removed and dissected for pathology, flow cytometry, immunoblot or qPCR assay. For pathology study, the brain slices were fixed in 4% paraformaldehyde (PFA) at 4˚C for 24 h, then processed in 30% sucrose/PBS (w/v) for 48 h before frozen in Tissue-Tek CRYO-OCT (Thermo Fisher Scientific, Waltham, MA, USA). For flow cytometry analysis, half of the brain was homogenized for further isolation and staining. For immunoblot and qPCR assay, the brain tissues were quickly frozen on dry ice and store at -80 ˚C for further usage.

Flow cytometry

Half of the brain from well-perfused mouse was dissected and dispersed in homogenization medium [RPMI1640 (Thermo Fisher Scientific) with 2% fetal bovine serum (FBS, R&D Systems, Minneapolis, MN, USA)] and passed through a disposable 70um strainer (Thermo Fisher Scientific) to remove debris. Cells were pelleted by centrifugation at 500g for 10 min at 4°C and resuspended in homogenization medium containing 25% percoll (Cytiva, Marlborough, MA, USA), then centrifuged at 520g for 20 min without break. After removal of the myelin layer and supernatant, cell pellets were washed twice by homogenization medium and filtered through 70um strainer, then centrifuged at 500g for 10 min at 4°C to pellet the cells. Cells were resuspended in PBS with 2% FBS and blocked with CD16/32 (1:100, Biolegend, San Diego, CA, USA) at room temperature for 10 min, then stained with antibodies as below: CD45-PerCP (1:200, Biolegend), CD11b-V450 (1:200, BD Biosciences, San Jose, CA, USA) at 4°C for 20min. After wash, the fluorescence signals were analyzed on Canto III (BD Biosciences).

Real-time Quantitative PCR Analysis (RT-qPCR)

Total RNA was extracted from midbrain with Trizol reagent (Thermo Fisher Scientific) according to the manufacturer's instructions. cDNA was transcribed with a SuperScript™ II Reverse Transcriptase (Thermo Fisher Scientific) according to the manufacturer's instructions. qPCR was performed using SYBR Green Super mix (Bio-Rad, Hercules, CA, USA) on an 7900HT Fast Real-Time PCR System (Applied Biosystems, Waltham, MA, USA). See **Supplemental Table 1** for primer sequences.

Western Blot

Brain tissues were homogenized and lysed in a prechilled radioimmunoprecipitation assay (RIPA) buffer (Millipore Sigma, Burlington, MA, USA) containing Protease Inhibitor Cocktail (Millipore Sigma) and Halt™ Phosphatase Inhibitor Cocktail (Thermo Fisher Scientific), followed by measurement of total protein concentration. Protein was loaded and separated by 4% stacking and 10% resolving SDS-PAGE gel. After transfer, polyvinylidene fluoride (PVDF) membrane (Millipore Sigma) was blotted with primary antibodies, including rabbit anti-phospho-S6 ribosomal protein (Ser235/236) (Cell Signaling Technology, Danvers, MA, USA), rabbit anti-LC3B (Novus Biologicals, Littleton, CO, USA), mouse anti-β-Actin (Millipore Sigma). After washing, species-appropriate secondary antibodies (IRDye 680RD- or 800CW-conjugated immunoglobulin G [IgG] (H+L) (Biosciences, Lincoln, NE, USA) was incubated for 1 h at a dilution of 1:5000. Odyssey imaging system (LI-COR Biosciences) was used to detect the signals. The gray density of blot bands was analyzed by ImageJ/Fiji (National Institutes of Health, Bethesda, MD, USA).

Immunofluorescent staining of tissues

Frozen sections were fixed with 4% PFA and permeabilized with 1× PBS containing 0.3% Triton X-100 for 30 min at room temperature, then treated with 50mM NH_4Cl in 1xPBS for 15 min. Sections were then blocked with 10% goat serum for 1 h at room temperature before staining with different combination of the following primary antibody overnight at 4 °C: rabbit anti-Phospho-S6 Ribosomal Protein (Ser235/236) (Cell Signaling), rat anti-Lamp1 (Santa Cruz Biotechnology, Dallas, TX, USA), mouse anti-Iba1 (Abcam), mouse anti-NeuN (Millipore Sigma), mouse anti-GFAP (Cell Signaling), mouse anti-Tmem119 (Proteintech, Rosemont, IL, USA), and followed by incubation for 1 h with appropriate Alexa Fluor secondary antibody (Thermo Fisher Scientific).

Slides were mounted with VECTASHIELD DAPI-containing medium (Vector Laboratories, Burlingame, CA, USA). Fluorescence signals were visualized and captured by Nikon Ti-2 microscope. Images were analyzed by NIS-Elements AR for p-S6 signals semi-quantitative analysis.

Supplemental Table 1 Primer sequences used in qPCR assay*.

Target ID	Forward Primer	Reverse Primer
Tmem119	TTCTTCCGGCAGTACGTGAT	CGAGGATGGGTAGTAGGCTG
Iba1	ACAGCAATGATGAGGATCTGC	CTCTAGGTGGGTCTTGGGAAC
Lamp1	CATCAGCAAAGAGATCTACACCAT	GAAGGTCCATCCTGTGTGCAG
Tnfa	GAAAGCATGATCCGCGACG	AGAGGGAGGCCATTTGGGAAC
Il1b	GAAATGCCACCTTTTGACAGTG	TGGATGCTCTCATCAGGACAG
Il6	CCAGAGATACAAAGAAATGATGG	ACTCCAGAAGACCAGAGGAAAT
Ccl2	TCACCTGCTGCTACTCATTCACCA	AGCACAGACCTCTCTCTTGAGCTT
Ccl5	GGCACACACTTGGCGGTTCCT	TGCCTACCTCTCCCTCGCGC
Cxcl9	GTGGAGTTCGAGGAACCCTAG	ATTGGGGCTTGGGGCAAAC
Cxcl10	GCCGTCATTTTCTGCCTCAT	GCTTCCCTATGGCCCTCATT
Cd86	TCAGATCAAGGACATGGGCTC	GTGACCTTGCTTAGACGTGC
Ifng	TCATGGCTGTTTCTGGCTGT	CACCATCCTTTTGCCAGTTCC
Nos2 (iNOS)	GCCCCGCTACTACTCCATCAG	AGCCACTGACACTTCGCACAA
Il4	AACGTCCTCACAGCAACGAA	AGGCATCGAAAAGCCCGAAA
Il10	TGAGGCGCTGTCATCGATTT	TGGCCTTGTAGACACCTTGG
Tgfb1	AGCTGCGCTTGCAGAGATTA	AGCCCTGTATTCCGTCTCCT
Ccl22	GACACCTGACGAGGACACAT	CCACATTGGCACCATAGGGA
Cd163	TGCTGTCACTAACGCTCCTG	TCATTCATGCTCCAGCCGTT
Cd204 (Msr1)	AGTGCTGTCTTCTTTACCAGCA	CTGAAGGGAGGGGCCATTTT
Cd206	GTGGAGTGATGGAACCCCAG	CTGTCCGCCCAGTATCCATC
Ym1	GAAGCTCTCCAGAAGCAATCC	GAGTACACAGGCAGGGGTCA
Tfrc	TCCGCTCGTGGAGACTACTT	ACATAGGGCGACAGGAAGTG
Retnla	AAGGAACTTCTTGCCAATCCAG	CTCCCAAGATCCACAGGCAAA
Tbp	TGCTGTTGGTGATTGTTGGT	CTGGCTTGTGTGGGAAAGAT

- All primers are shown as 5′ to 3′.
