# Supplementary material for: A multifaceted evaluation of microgliosis and differential cellular dysregulation of mammalian target of rapamycin signaling in neuronopathic Gaucher disease
Source: Front Mol Neurosci. 2022 Sep 20;15:944883. doi: 10.3389/fnmol.2022.944883 (PMC9530712; doi:10.3389/fnmol.2022.944883)
Supplement: Supplementary file 2 [file Data_Sheet_2.pdf]

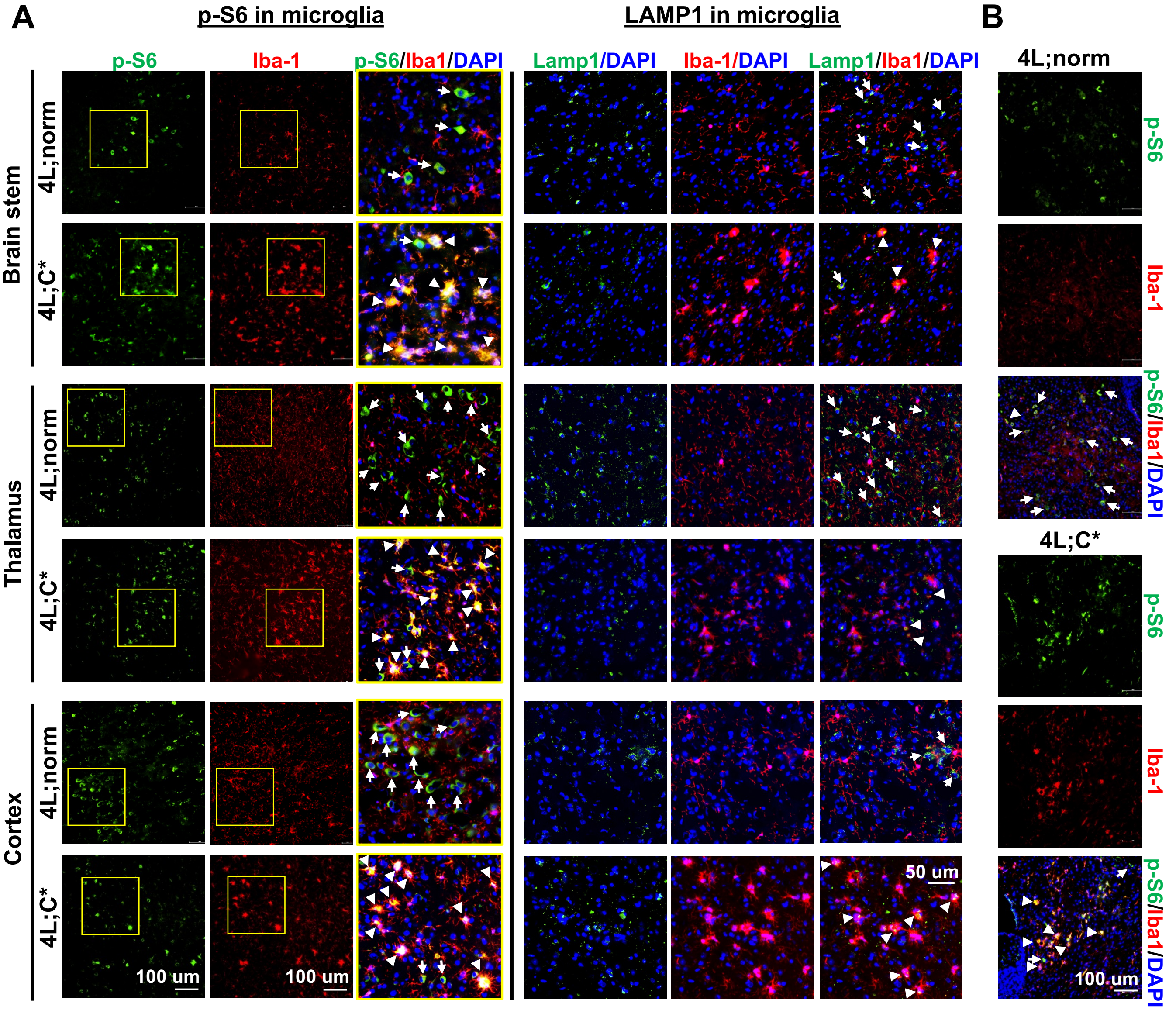

Supplemental Figure S1. pS6 and Lamp1 expression in microglia of additional brain regions. (A) Increased expression of p-S6 and Lamp1 in activated microglia of 4L;C* mice. The areas in yellow squares are enlarged and shown. White triangles indicate for p-S6$^+$Iba1$^+$ cells (in left panel) or Lamp1$^+$Iba1$^+$ cells (in right panel). White arrows indicate for p-S6$^+$Iba1$^-$ cells (in left panel) or Lamp1$^+$Iba1$^-$ cells (in right panel). **(B)** Increased p-S6 expression in activated microglia of deep cerebellar nuclei region of 4L;C* mice as compared to 4L;norm mice. White triangles indicate for p-S6$^+$Iba1$^+$ cells and white arrows for p-S6$^+$Iba1$^-$ cells.

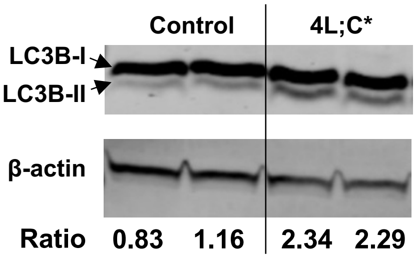

Supplemental Figure S2. Evaluation of LC3B protein levels by Western blot analysis. Midbrain samples were used for the experiment. The intensities of LC3B-II bands were analyzed by Image J/Fiji and normalized by those of β-actin as the internal control. Ratios are shown as fold changes of LC3B-II in 4L;C* over those in normal controls.

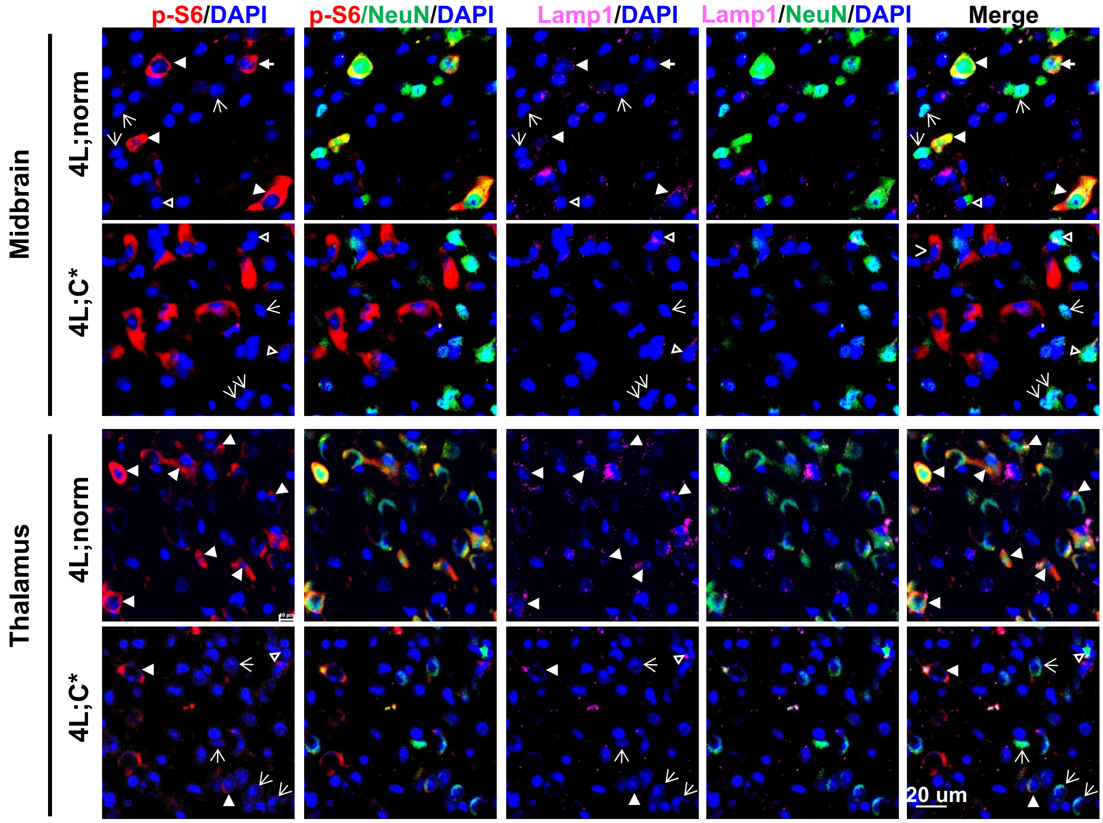

Supplemental Figure S3. Expression of p-S6 and Lamp1 in neurons of midbrain and thalamus regions. Representative views are shown, with white triangles indicating for S6^{+}Lamp1^{+} neurons, open triangles for S6^{-}Lamp1^{+} neurons, solid arrows for S6^{+}Lamp1^{-} neurons and open arrows for S6^{-}Lamp1^{-} neurons.

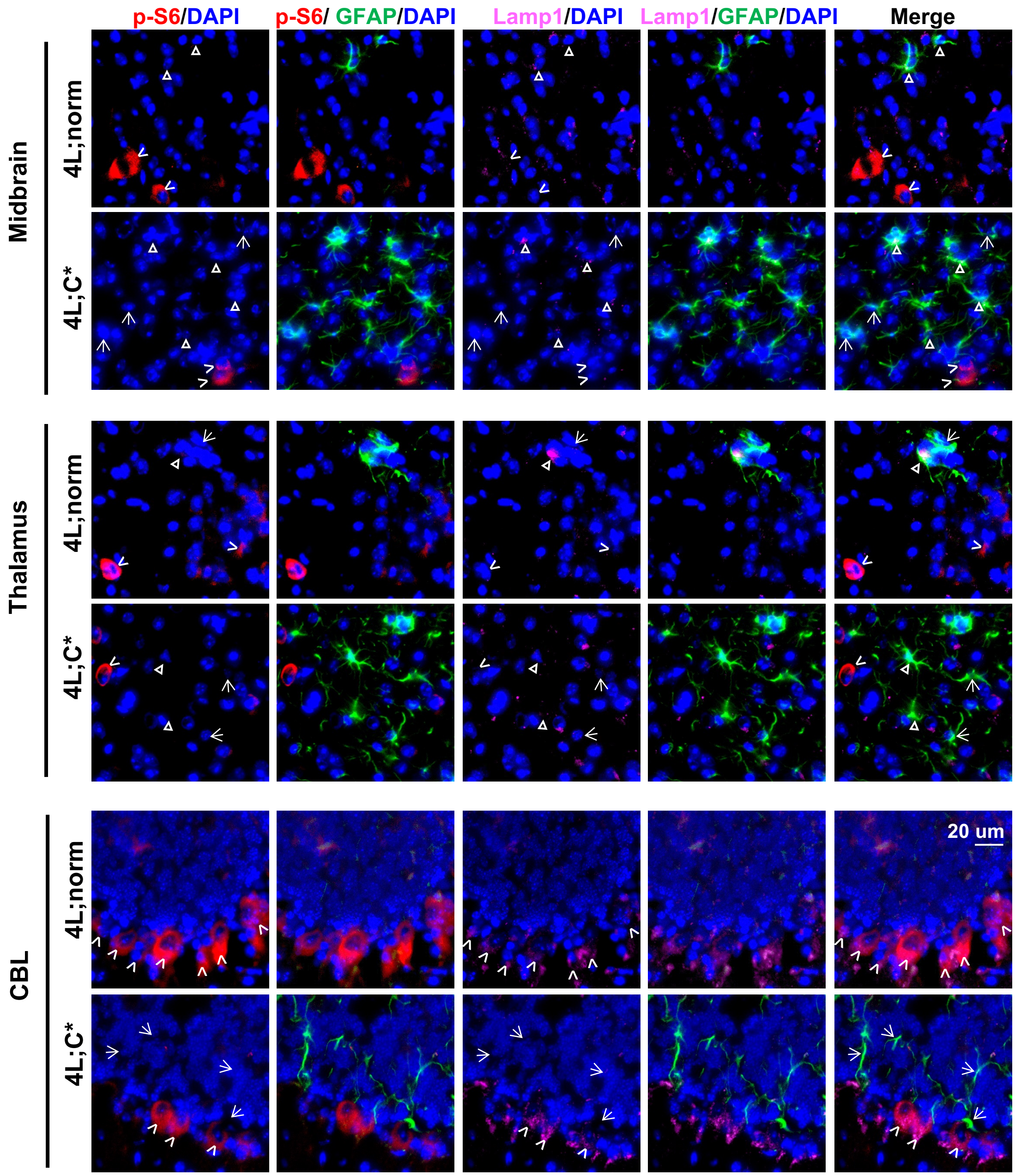

Supplemental Figure S4. Expression of p-S6 and Lamp1 in astrocytes of midbrain, thalamus and CBL regions. Representative views are shown, with open triangles indicating for $S6^{-}Lamp1^{+}$ astrocytes, open arrows for $S6^{-}Lamp1^{-}$ astrocytes and arrow heads for $S6^{+}Lamp1^{+/-}$ non-astrocytic cells.
